# Effects of HIV on executive function and verbal fluency in Cameroon

**DOI:** 10.1038/s41598-018-36193-7

**Published:** 2018-12-12

**Authors:** Georgette D. Kanmogne, Julius Y. Fonsah, Bin Tang, Roland F. Doh, Anne M. Kengne, Anya Umlauf, Claude T. Tagny, Emilienne Nchindap, Léopoldine Kenmogne, Donald Franklin, Dora M. Njamnshi, Dora Mbanya, Alfred K. Njamnshi, Robert K. Heaton

**Affiliations:** 1 Department of Pharmacology and Experimental Neuroscience, College of Medicine, University of Nebraska Medical Center, Omaha, NE 68198 USA; 2 Faculty of Medicine and Biomedical Sciences, University of Yaoundé I, Yaoundé, Cameroon; 3 Department of Neurology, Yaoundé Central Hospital/Brain Research Africa Initiative (BRAIN), Yaoundé, Cameroon; 4 Department of Psychiatry, University of California San Diego, 9500 Gilman Drive, La Jolla, CA 92093 USA; 5 Yaoundé University Teaching Hospital, Yaoundé, Cameroon; 6 HIV-Day Care Service, Yaoundé Central Hospital, Yaoundé, Cameroon

## Abstract

HIV-associated neurocognitive disorders (HAND) are frequently associated with impaired executive function and verbal fluency. Given limited knowledge concerning HAND in Sub-Saharan-Africa and lack of Cameroonian adult neuropsychological (NP) test norms, we administered four executive function [Halstead Category Test (HCT), Wisconsin Card Sorting Test (WCST), Color Trails-II (CTT2), and Stroop Color-Word-Interference (SCWT)] and three verbal fluency (Category, Action, and Letter Fluency) tests to 742 adult Cameroonians (395 HIV−, 347 HIV+). We developed demographically-corrected NP test norms and examined the effects of HIV and related variables on subjects’ executive function and verbal fluency. HIV+ subjects had significantly lower T-scores on CTT2 (P = 0.005), HCT (P = 0.032), WCST (P < 0.001); lower executive function composite (P = 0.002) and Action Fluency (P = 0.03) T-scores. ART, viremia, and CD4 counts did not affect T-scores. Compared to cases harboring other viral subtypes, subjects harboring HIV-1 CRF02_AG had marginally higher CTT2 T-scores, significantly higher SCWT (P = 0.015) and executive function (P = 0.018) T-scores. Thus, HIV-1 infection in Cameroon is associated with impaired executive function and some aspects of verbal fluency, and viral genotype influenced executive function. We report the first normative data for assessing executive function and verbal fluency in adult Cameroonians and provide regression-based formulas for computing demographically-adjusted T-scores. These norms will be useful for investigating HIV/AIDS and other diseases affecting cognitive functioning in Cameroon.

## Introduction

HIV enters the CNS in the early stages of infection, where it productively infects brain macrophages and microglia and can induce injury and dysfunction of neurons and other CNS cells^1,2^. These brain pathologies frequently result in behavioral, motor and cognitive abnormalities referred to as HIV-associated neurocognitive disorders (HAND)^2–6^. Studies in Western countries show that although combination antiretroviral therapy (cART) use has reduced the prevalence of HIV-associated dementia (the most severe form of HAND), the prevalence of milder forms of HAND such as asymptomatic neurocognitive impairment (ANI) and mild neurocognitive disorders (MND) have not improved in the cART era^4–10^.

Our current knowledge of HAND prevalence and pathogenesis is mostly derived from studies performed in Western countries, using subjects infected with subtype-B HIV-1^2,4–8,11,12^. Since the beginning of the HIV/AIDS epidemic over 3 decades ago, Sub-Saharan Africa (SSA) has consistently been the hardest hit region in the world, and most of the 35 million people who have died from HIV/AIDS-related illnesses since the start of the epidemic were in SSA. Of the 36.7 million individuals worldwide currently living with HIV/AIDS, 70% are in SSA^13^, many with non-B viral subtypes, and there is little information concerning whether these individuals are at risk for cognitive and neurological complications.

Like most countries in SSA, Cameroon, a country of about 25 million inhabitants^14^, still has a heavy HIV/AIDS burden. According to recent UNAIDS estimates, the prevalence of HIV infection in the general adult population in Cameroon is 3.8%, with a prevalence of 5.1% among adult females, 2.5% among adult males, 24.3% among female sex workers, and 37.2% among men who have sex with men^15^. The HIV epidemic in Cameroon is further characterized by a high viral genetic diversity, with circulating strains including several group M HIV-1 subtypes, HIV-1 groups O and N, circulating recombinant forms and unique recombinant forms^16–18^. Therefore, it is important to understand the neurological and neurocognitive effects of the various forms of HIV infection in this country.

It has been shown that the cognitive domains most likely impaired in HIV/AIDS patients include executive function^3,5,19–22^ and verbal fluency^5,23,24^. The executive function domain includes cognitive abilities involved in life tasks such as planning, organizing and strategizing, management, paying attention, mental control, and self-regulation^25,26^. The verbal fluency domain includes cognitive abilities involved in language and semantic memory, including word knowledge and retrieval^27–29^. Executive function and verbal fluency are both supported by the frontal lobes, and impairments in these cognitive domains correlate with damage to frontal brain systems^27,30–32^. These cognitive abilities are assessed using neuropsychological (NP) tests. However, using NP tests to assess cognitive abilities in any given population requires normative data appropriate to that population, in order to ensure validity, accurate classification and clinical diagnoses. Some norms for cognitive evaluation of children were previously reported in Cameroon and used to assess cognitive function in children with sickle cell disease^33,34^. Currently there are no adult Cameroonian norms for assessing executive function or verbal fluency. Our current study establishes normative scores for four commonly used NP tests of executive function: the Halstead Category Test (HCT), Wisconsin Card Sorting Test (WCST), Color Trails-II test (CTT2), and Stroop Color-Word Interference test (SCWT)^25,35–39^; and three commonly used NP tests of verbal fluency: Category Fluency, Action Fluency, and Letter Fluency^27–30^. We adjusted the data for demographic factors (age, gender, and education), and further assessed the effects of HIV infection, immune status, ART, viremia and viral genotype on subjects’ performance on these NP tests.

## Results

### Demographic and laboratory characteristics

In 2016, an estimated 560,000 Cameroonians were living with HIV/AIDS, and 29,000 HIV/AIDS-related deaths were recorded^14,15^. Females represented about 65% of HIV-infected adults (15 to 49 years old), and 70% of HIV-infected youths and younger adults (15 to 24 years old)^14,15^. A total of 742 subjects were recruited for this study, including 395 HIV-seronegative controls and 347 HIV+ cases. Overall, HIV+ subjects were somewhat older, less educated, and had a smaller proportion of males than the control group (Table 1). The median CD4 cell counts in the HIV+ cohort was 407 (IQR 246, 574) cells/µl. For the 173 cases with detectable viral load, the mean log viral load was 4.59 ± 1.28 log copies/ml. For the 343 cases with known treatment status, 189 (55.1%) were on cART, of whom 139 (73.5%) had undetectable viral load; 148 (43.1%) were treatment naïve, of whom 34 (23%) had undetectable viral load (<50 copies/mL) (Table 1). Six cases (1.75%) had stopped cART and/or took cART only for a short period (e.g. during pregnancy) (Table 1). Many subjects could not complete the neuromedical, NP battery, and lab tests on the same day, and had to return to the hospital on a different day for specimen collection and lab testing. Some of those subjects did not return for lab testing, resulting in 9.5% missing lab data. Additionally, 7 participants (2%) had missing CD4 (3 participants) or viral load (4 participants), which apparently was due to the relevant equipment not being fully operational at the time of specimen collection.

**Table 1:** Demographic and clinical characteristics by HIV status. Values are Mean (SD), Median [IQR], or N (%).

Characteristics	HIV−		HIV+		P Value
	N^a^	Mean (SD) or N (%)	N^a^	Mean (SD), Median [IQR], or N (%)	
DEMOGRAPHICS					
Age (years)	395	34.6 (10.5)	347	37.9 (9.38)	<0.001
Education (years)	394	12.4 (4.23)	346	9.65 (3.78)	<0.001
Male, N (%)	395	137 (34.7%)	347	77 (22.2%)	<0.001
HIV DISEASE					
CD4	—	—	306	407 [246, 574]	—
Viral Load, N (%)			305		
Undetectable				173 (56.7%)	
Detectable				132 (43.3%)	
Log10 Viral Load (among subjects with detectable VL)	—	—	173	4.59 (1.28)	—
ART Status, N (%)			343		
ART	—	—	—	189 (55.1%)	—
Naive	—	—	—	148 (43.1%)	—
Not Current	—	—	—	5 (1.46%)	—
Other (1 ZIDOVIR in pregnancy only, and 1 Vanhivax)	—	—	—	1 (0.29%)	—

Notes: Student’s t-test was applied for continuous variables, and Fisher’s exact test for categorical variables; SD, standard deviation; IQR, interquartile range. ^a^Total number of participants with available data for the corresponding variable.

### Raw scores and standardized scores

Raw scores were converted to scaled scores (SS) as detailed in the Methods section, and Table 2 shows the SS and corresponding raw scores for CTT2 (time), HCT (total errors), SWCT (total correct), WCST (total errors), Category Fluency, Action Fluency, and Letter Fluency. Table 3 shows the equations used to calculate demographically-corrected T-scores for executive function (CTT2, HCT, SCWT, and WCST) and verbal fluency (Category Fluency, Action Fluency, and Letter Fluency) tests, using regression-based analyses. For both samples (HIV− and HIV+) robust age and education effects were seen on raw scores for all the tests, and for gender on most tests. In every case (all tests for both samples), education and gender effects were absent in the corrected T-scores. Age effects were fully controlled on T-scores for the HIV− samples, and either fully controlled (CTT2, WCST, Executive Function composite) or greatly attenuated in the T-scores of the HIV+ sample. All significant HIV effects on T-scores and deficit scores remained significant if age was covaried in the relevant analyses.

**Table 2:** Conversion of the raw scores to scaled scores for tests assessing executive function and verbal fluency domains.

Scaled Score	Executive Function				Verbal Fluency			Scaled Score
	Color Trails II (Time)	HCT (Total Errors)	SCWT (Total Correct)	WCST (Total Errors)	Category Fluency Trial 1 Words	Action Fluency	Letter Fluency	
1	429–450	163–208	0–7	60–64	0–2	—	—	1
2	396–428	152–162	8–12	53–59	3–4	—	0	2
3	329–395	143–151	13–16	51–52	5	0–2	1	3
4	269–328	133–142	17–18	50	6	3–4	2–8	4
5	234–268	122–132	19–20	48–49	7	5	9–11	5
6	199–233	111–121	21–23	45–47	8–9	6	12–14	6
7	163–198	100–110	24–25	40–44	10	7–8	15–17	7
8	143–162	91–99	26–29	36–39	11–12	9	18–21	8
9	127–142	80–90	30–32	30–35	13–14	10–11	22–26	9
10	113–126	69–79	33–36	26–29	15	12	27–31	10
11	101–112	55–68	37–40	21–25	16–17	13–14	32–34	11
12	91–100	43–54	41–44	17–20	18–19	15–16	35–38	12
13	80–90	34–42	45–48	14–16	20–21	17–18	39–44	13
14	72–79	25–33	49–53	12–13	22–23	19–20	45–47	14
15	65–71	22–24	54–60	11	24–25	21–22	48–50	15
16	55–64	19–21	61–71	9–10	26–27	23–25	51–56	16
17	49–54	17–18	72–82	8	28	26–33	57–71	17
18	11–48	14–16	83–122	6–7	29–41	34–35	72–77	18
19	0–10	0–13	123–133	0–5	42–45	36–45	78–80	19

**Table 3:** Demographically-corrected T-score calculation formulas based on scaled scores for tests assessing executive function and verbal fluency domains.

Test	Formula
Executive Function	
Color Trails II Time	50 + 10*[(scaled score) − (9.359 + 2.690*((edu + 1)/10) − 8.673*(age/100) − 0.029*male)]/2.485
HCT Total Errors	50 + 10*[(scaled score) − (9.640 + 1.944*((edu + 1)/10) − 7.529*(age/100) + 0.999*male)]/2.639
Stroop Interference	50 + 10*[(scaled score) − (10.556 + 1.464*((edu + 1)/10) − 7.586*(age/100) + 0.064*male)]/2.755
WCST Total Errors	50 + 10*[(scaled score) − (8.230 + 1.958*((edu + 1)/10) − 3.361*(age/100) + 0.816*male)]/2.835
Verbal Fluency	
Category Fluency Trial 1 Words	50 + 10*[(scaled score) − (8.710 + 2.454*((edu + 1)/10) − 6.271*(age/100) + 0.320*male)]/2.665
Action Fluency	50 + 10*[(scaled score) − (8.144 + 2.406*((edu + 1)/10)−4.400*(age/100) + 0.475*male)]/2.616
Letter Fluency	50 + 10*[(scaled score) − (5.736 + 3.819*((edu + 1)/10) − 3.040*(age/100) + 0.519*male)]/2.440

Abbreviation: edu = education; male = 1 for male, 0 for female.

### Effects of HIV infection on executive function

Analyses revealed that compared to controls, HIV+ subjects had significantly lower T-scores on CTT2, HCT total errors, and WCST total errors (Table 4). There was no group difference in Stroop Interference T-scores, but a significantly lower executive function composite T-score was seen for HIV+ subjects compared to seronegative controls (Table 4).

**Table 4:** Comparisons of executive function and verbal fluency demographically-corrected T-scores between controls and HIV+ patients. Notes: Cohen’s d compares HIV+ to HIV-; Multiple testing was not corrected; The higher the T-score, the better is NP performance. SD, standard deviation; CI, confidence interval; HCT, Halstead Category Test; WCST, Wisconsin Card Sorting Test.

Test	HIV− (N = 395)		HIV+ (N = 347)		Cohen’s d (95% CI)	P Value
	N	Mean (SD)	N	Mean (SD)		
Executive Function						
Color Trails II Time	362	50.0 (10.0)	323	47.6 (11.8)	−0.21 (−0.37, −0.06)	0.005
HCT Total Errors	355	50.0 (9.98)	308	48.3 (9.69)	−0.17 (−0.32, −0.01)	0.032
Stroop Interference	362	50.0 (10.0)	316	49.6 (10.7)	−0.04 (−0.19, 0.11)	0.62
WCST Total Errors	351	50.0 (10.0)	308	46.1 (9.87)	−0.39 (−0.54, −0.23)	<0.001
Executive Function Summary Score	317	49.7 (6.73)	272	48.0 (6.75)	−0.26 (−0.42, −0.10)	0.002
Verbal Fluency						
Category Fluency	364	50.0 (10.0)	322	49.9 (9.88)	−0.01 (−0.16, 0.14)	0.89
Action Fluency	364	50.0 (9.98)	322	48.2 (11.7)	−0.16 (−0.31, −0.01)	0.031
Letter Fluency	364	50.0 (9.99)	321	49.3 (9.72)	−0.07 (−0.22, 0.08)	0.37
Verbal Fluency Summary Score	364	50.0 (8.03)	320	49.1 (8.60)	−0.11 (−0.26, 0.05)	0.16

Analyses of the degree of impairment in executive functioning showed that compared to HIV− controls, a significantly higher proportion of HIV+ subjects showed impairment on the CTT2 (odds ratio (OR) 2.53) and WCST (OR:1.94) (P < 0.001, Table 5). There was no significant difference between the two groups in impairment rates on the HCT and SCWT, but analysis of the composite executive function domain deficit score showed that the proportion of HIV+ subjects with impairment in executive functioning (20.2%) was almost double the proportion seen in HIV− controls (12%) (P = 0.007, Table 5).

**Table 5:** Comparisons of the proportions of impairment in executive function and verbal fluency domains between controls and HIV+ patients.

Test	HIV− (N = 395)	HIV+ (N = 347)	OR (95% CI)	P Value
Executive Function, impaired, N (%)				
Color Trails II Time	46 (12.7%)	87 (26.9%)	2.53 (1.68, 3.85)	<0.001
HCT Total Errors	51 (14.4%)	53 (17.2%)	1.24 (0.80, 1.93)	0.34
Stroop Interference	51 (14.1%)	50 (15.8%)	1.15 (0.73, 1.79)	0.59
WCST Total Errors	53 (15.1%)	79 (25.6%)	1.94 (1.29, 2.92)	0.001
Executive Function Domain	38 (12.0%)	55 (20.2%)	1.86 (1.16, 3.00)	0.007
Verbal Fluency, impaired, N (%)				
Category Fluency Trial 1 Words	50 (13.7%)	47 (14.6%)	1.07 (0.68, 1.69)	0.83
Action Fluency	51 (14.0%)	79 (24.5%)	1.99 (1.33, 3.01)	0.001
Letter Fluency	47 (12.9%)	54 (16.8%)	1.36 (0.87, 2.13)	0.16
Verbal Fluency Domain	47 (12.9%)	76 (23.8%)	2.10 (1.38, 3.21)	<0.001

Notes: OR, odds ratio, compares HIV+ to HIV−; CI, confidence interval; HCT, Halstead Category Test; WCST, Wisconsin Card Sorting Test; Impaired, domain deficit score > 0.5 or individual test deficit score >= 1.

### Effects of HIV infection on verbal fluency

Compared to controls, HIV+ subjects had significantly lower T-scores in the test of Action Fluency (P = 0.03), but there was no difference between the two groups on Category Fluency, Letter Fluency, or the composite verbal fluency T-scores (Table 4). Analyses of the prevalence of impairment in verbal fluency showed that compared to HIV− controls, a significantly higher proportion of HIV+ subjects had impairment in Action Fluency (P = 0.001), and again there was no significant difference between the two groups on Category Fluency or Letter Fluency (Table 5). However, the analysis that considered all tests of the verbal fluency domain together (domain deficit score) showed impairment in 23.8% of HIV+ subjects, compared to 12.9% for the HIV− controls group (P < 0.001, Table 5).

### Effects of viremia on executive function and verbal fluency

To determine whether viral load affected executive functioning and/or verbal fluency in HIV+ participants, we compared T-scores of those with undetectable (<50 viral copies/ml, n = 173) and detectable (≥50 copies/ml, n = 132) viral loads. Data showed no significant difference in CTT2, HCT, SCWT, and WCST T-scores between the two groups, and no difference in the overall executive function composite score between virally suppressed cases (undetectable) and cases with detectable viral loads (*d*: 0.03; 95% CI: −0.28, 0.22, P = 0.81). Additional analyses comparing cases having undetectable viral loads (<50 copies/ml, n = 173) with participants having viral loads >50 and <100,000 copies/ml (n = 80), and cases with very high viral loads (≥ 100,000 copies/ml) (n = 51) also showed no group differences on individual executive function scores, and no difference on the overall executive function composite T-score. Data also revealed no effect of viral loads on Category Fluency, Action Fluency, or Letter Fluency T-scores, or the overall verbal fluency T-scores.

### Effects of cART on executive function and verbal fluency

To determine whether antiretroviral treatment could affect patients executive functioning and/or verbal fluency, we performed comparative analyses of T-scores of HIV+ subjects who were treatment naïve and those on cART. Analyses showed that patients on cART had marginally higher SCWT T-scores compared to participants not on cART (*d*: 0.19; 95% CI: 0.03, 0.42, P = 0.09), but no differences were found on CTT2, HCT, and WCST T-scores, and no difference in the overall executive function composite T-score between subjects not on treatment and those on cART. Similarly, cART had no effect on Category Fluency, Action Fluency, or Letter Fluency T-scores, and no effect on the overall verbal fluency composite T-scores, although marginal significance was observed for Letter Fluency and verbal fluency composite T-scores (*d*: 0.20; 95% CI: −0.02, 0.43, P = 0.07 and *d*: 0.19; 95% CI: −0.03, 0.41, P = 0.09, respectively).

Of the 189 cases on cART, 177 (93.65%) were on first line regimens; only 12 (6.3%) had been on regimens that included a second line cART: 2 nucleoside/nucleotide reverse transcriptase inhibitors (NRTIs) or 2NRTIs + 1 non-nucleoside reverse transcriptase inhibitors (NNRT), plus Lopinavir/Ritonavir (LPV/r, n = 7), Atazanavir/Ritonavir (ATV/r, n = 3), or Darunavir/Ritonavir (DRV/r n = 2). Of the 177 cases on first line regimens, 123 (69.5%) were on Lamivudine (3TC) + Zidovudine (ZDV)+ Nevirapine (NPV) or Efavirenz (EFV); and 64 (36.16%) were on 3TC + Tenofovir (TDF) + NVP or EFV. We performed additional analyses of cases on regimens containing NVP (n = 91) or ZDV (n = 68 to 75) and cases on regimens that did not contain NVP (non-NVP, n = 47 to 58) or ZDV (non-ZDV, n = 55 to 64). Compared to the non-NVP group, use of NVP was associated with marginally higher T-scores on CTT2 (*d*: 0.32; 95% CI: −0.026, 0.67, P = 0.07). There were no significant differences in HCT, SCWT, and WCST T-scores, and no difference in the overall executive function composite T-scores between the non-NVP and NVP groups. There were no differences in the Category Fluency, Action Fluency, or Letter Fluency T-scores, or the overall verbal fluency composite T-scores of the non-NVP and NVP groups. There were no significant differences in CTT2, HCT, SCWT, and WCST T-scores, or the overall executive function composite T-scores of the ZDV and non-ZDV groups; no differences in the Category Fluency, Action Fluency, or Letter Fluency T-scores, or the overall verbal fluency composite T-scores of the ZDV and non-ZDV groups.

Compared to cases that had been on multiple (≥2) cART regimens (n = 46 to 51), cases that been on only one cART regimen (n = 110 to 125) had marginally higher HCT T-scores (*d*: 0.31; 95% CI: −0.01, 0.64, P = 0.06). There were no differences on CCT2, SCWT, and WCST T-scores, or the overall executive function composite T-scores of cases that had only one cART regimen and those that had been on multiple cART regimens. There were no differences in the Category Fluency, Action Fluency, or Letter Fluency T-scores, or the overall verbal fluency composite T-scores of cases that had been on only one regimen and those that had been on multiple cART regimens.

### Effects of the immune system on executive function and verbal fluency

To determine whether the immune status could affect executive functioning or verbal fluency among Cameroonian subjects, we compared analyses of T-scores of HIV+ subjects with low (<350 cells/µl) and higher (≥350 cells/µl) CD4 cell counts. Analyses showed no significant difference in CTT2 (*d*: 0.12; 95% CI: −0.11, 0.36, P = 0.30), HCT (*d*: 0.18; 95% CI: −0.06, 0.42, P = 0.15), SCWT (*d*: 0.12; 95% CI: −0.12, 0.36, P = 0.32), WCST (*d*: 0.18; 95% CI: −0.06, 0.42, P = 0.14), or executive function composite T-scores (*d*: 0.20; 95% CI: −0.05, 0.46, P = 0.12).

Analyses showed no significant difference in Category Fluency (*d*: 0.06; 95% CI: −0.18, 0.30, P = 0.63), Action Fluency (*d*: 0.05; 95% CI: −0.18, 0.29, P = 0.65), Letter Fluency (*d*: 0.15; 95% CI: −0.08, 0.39, P = 0.22), or the summary verbal fluency (*d*: 0.10; 95% CI: −0.14, 0.33, P = 0.42) T-scores of cases with lower and those with higher CD4 cell counts.

### Genetic diversity of Cameroon HIV isolates

We successfully amplified the protease (PR), reverse transcriptase (RT), group specific antigen (gag), envelope (env, C2V3), transactivator of transcription (tat), and/or negative regulatory factor (nef) genes in plasma samples from 161 HIV+ Cameroonians. Combined analyses of the PR, RT, gag, env, tat, and/or nef sequences showed that HIV-1 CRF02_AG was the predominant viral subtype, with 95 (59%) of the 161 subjects infected with this viral strain. Genetic analyses of PR, RT, gag, env, tat, and/or nef sequences also showed multiple genetic recombinants. Twenty four subjects (14.9%) harbored viruses that had CRF02_AG genotype in some viral gene regions, and a different genotype in other regions: 4 CRF02_AG/F2, 3 CRF02_AG/CRF01_AE, 3 CRF02_AG/D, 2 CRF02_AG/CRF11_cpx, 2 G/CRF02_AG, 2 CRF02_AG/CRF22_01A1, and 1 subject each for CRF22_01A1/CRF02_AG/CRF01_AE, CRF02_AG/A1, F1/CRF02_AG, A1/AD/CRF02_AG/CRF09_cpx, CRF02_AG/CRF19_cpx, CRF02_AG/CRF18_cpx, and U(unclassified)/D/CRF02_AG. Forty two subjects (26.1%) were infected with non-CRF02_AG strains, including subjects harboring viruses of a specific subtype in one or more of the six gene regions analyzed, and a different subtype in other regions: 7 CRF37_cpx, 7 CRF11_cpx; 3 each for CRF13_cpx, CRF18_cpx, CRF01_AE, subtype G, subtype F2, and subtype D; 1 each for G/A1, CRF11_cpx/A1, CRF01_AE/CRF22_01A1, A1/H, CRF22_01A2/CRF01_AE/CRF22_01A1, CRF19_cpx, CRF37_cpx/A1, G/CRF11_cpx, A1/A2/CRF01_AE, CRF22_01A1.

### Effects of HIV genotype on executive function and verbal fluency

To explore whether viral genotype may influence subjects’ executive functioning or verbal fluency, we performed comparative analyses of T-scores of cases infected with HIV-1 CRF02_AG (AG), the predominant subtype in Cameroon, and cases infected with non-CRF02_AG viruses (non-AG) or viruses that had CRF02_AG genotype in some of the 6 gene regions analyzed and different genotypes in other gene regions (AG-Plus). Compared to AG subjects, non-AG and AG-Plus subjects had marginally lower T-scores on CTT2 (*d*: 0.31; 95% CI: −0.02, 0.64, P = 0.058) and significantly lower T-scores on SCWT (*d*: 0.41; 95% CI: 0.08, 0.74, P = 0.015). There were no significant differences between the two groups on the HCT (*d*: 0.11; 95% CI: −0.23, 0.45, P = 0.53) and WCST (*d*: 0.18; 95% CI: −0.15, 0.52, P = 0.29) T-scores. Analysis of the composite executive function T-scores showed that compared to AG subjects, non-AG and AG-Plus subjects had significantly lower overall executive function composite T-scores (*d*: 0.43; 95% CI: 0.07, 0.78, P = 0.018). There was no difference between the two groups on Category Fluency, Action Fluency, or Letter Fluency T-scores, or the verbal fluency summary T-scores.

## Discussion

Performances on neurocognitive tests are influenced by population demographics, language, and cultural backgrounds^6,40,41^. Therefore, it is important to have population-appropriate, demographically-corrected normative standards to permit validity, accurate classification, and clinical diagnoses of neurocognitive disorders^11^. The present study provides the first reported adult normative data for assessing executive function and verbal fluency in Cameroon. It provides demographically-corrected normative scores based upon results of healthy HIV− controls for four tests of executive function (CTT2, HCT, WCST, and SCWT) and three tests of verbal fluency (Category Fluency, Action Fluency, and Letter Fluency), as well as demographically-corrected norms for executive function and verbal fluency composites. We further performed comparative analyses of executive functioning and verbal fluency between the HIV− controls and HIV+ groups. Analyses using demographically corrected standardized scores showed significant effects of HIV infection on executive function, with HIV+ subjects having significantly lower T-scores on CTT2, HCT, and WCST compared to the control group; and 26.9% and 25.6% of cases showing deficits on CTT2 and WCST compared to 12.7 and 15.1% of controls. Combining data for all four executive function tests further showed significantly higher proportion of cases with impairment in executive functioning (20.2%) compared to controls (12%). These findings are in agreement with previous studies in both developed^4–8^ and resource-limited^9,42–46^ countries showing that HIV infection was associated with impairments in executive function, including studies in Uganda^42–44^, Botswana^45^, and Nigeria^46^.

Of the three NP tests assessing the verbal fluency domain, the Action Fluency test was more sensitive for detecting differences between cases and controls, with 24.5% of cases showing impairment in Action Fluency compared to 14% of controls (P = 0.001). Other studies previously showed greater impairment in Action Fluency than in Category Fluency among HIV+ subjects^47,48^; also compared to infected subjects with deficits in Category Fluency or Letter Fluency, deficits in Action Fluency in HIV+ subjects is associated with larger decline in activities of daily living^49^ and increased levels of astrocytosis markers in the cerebrospinal fluid^50^.

Other studies in SSA, including in South Africa^51^, Uganda^44^, and Nigeria^46^ also found an HIV effect on verbal fluency, with the three tests of verbal fluency showing differential sensitivity. The South African study showed significant deficits in Letter Fluency and Action Fluency among HIV+ subjects compared to HIV− controls, and no difference in Category (animal) Fluency^51^; whereas the Ugandan study showed significant HIV-related deficits in Category (animal recall) Fluency^44^, and another study in Nigeria reported no significant differences at all in Verbal Fluency between HIV+ subjects and seronegative controls^52^. This suggests that the sensitivity of each of these 3 tests of verbal fluency may vary based on populations and cultural backgrounds. However, in most of these studies, analyses of summary verbal fluency scores showed a significant deficit in verbal fluency among HIV+ subjects compared to HIV− controls^44,46,51^. This agrees with our current findings showing that, after correction for demographic variables (age, education, gender), a significantly higher proportion of HIV+ subjects had deficits in the verbal fluency domain (23.8%) compared to HIV− controls (12.9%).

Verbal fluency tests assess individuals’ abilities to correctly search and retrieve a limited set of words^28,29^. Like executive function tests, they require planning, organization, flexibility and decision-making. Coordination with different brain areas, including the frontal and temporal systems, is required to correctly execute these tests^27–30^. As a measure of language and executive function, Action Fluency is most associated with frontal brain systems whereas Letter Fluency is associated with different networks of the brain frontal region, and Category Fluency more with temporo-parietal areas^27,30,53,54^. The fact that HIV-infected subjects in our current study showed deficits in both executive function and Action Fluency suggests that HIV infection may especially cause frontal system dysfunctions in the Cameroonian population.

It is well known that psychoactive substances and social drugs such as alcohol and nicotine can affect neurocognitive functioning, and previous studies in Cameroon showed that for HIV+ subjects, alcohol use and smoking were associated with increased viral loads and oxidative stress^55,56^. It is unlikely that such confounds could have influenced our current data. As detailed in our inclusion/exclusion criteria in the Methods section, we screened all subjects for social drugs (alcohol and nicotine-cotinine) and twelve other psychoactive substances, including cocaine, oxycodone, opiates, barbiturates, marijuana (tetrahydrocannabinol, THC), and methamphetamine. No subject tested positive for cocaine, oxycodone, or opiates, and breathalyzer tests showed that no subject tested had alcohol in their system. Only one subject (HIV+) tested positive for methamphetamine; 3 (2 HIV+ and 1 HIV−) tested positive for THC, 4 (3 HIV+ and 1 HIV−) tested positive for barbiturates, and 17 (2.3%) (8 HIV+ and 9 HIV−) tested positive for nicotine-cotinine. These low numbers make it quite unlikely that substance use/abuse was a confounding factor in our current analyses.

Our current study showed that use of cART was associated with marginally higher SCWT T-scores. However, there was no effect of cART or viral loads on other tests of executive function, or on the executive function summary T-score, or verbal fluency (Category Fluency, Action Fluency, and Letter Fluency, or the verbal fluency summary T-score). NVP and EFV were the NNRTIs used in cART regimens, and use of regimens containing NVP was associated with marginally higher T-scores on CTT2. These findings suggest that non-NVP (EFV) regimens may have been slightly more likely to negatively affect at least some aspects of executive function, which corroborate other literature evidences showing that EFV is neurotoxic and is associated with increased risk of CNS adverse events and neurocognitive impairment^57–60^. We previously showed increased prevalence of depressive symptoms among HIV-infected Cameroonians^61^, showed that changes in cART regimens were associated with increased risk of non-adherence to treatment and that the presence of depressive symptoms correlated with non-adherence to cART^62^. Our current data also showed that, compared to subjects who had been on only one cART regimen, use of multiple cART regimens or changes in treatment regimens was associated with poorer performance in HCT. Changes in cART regimens are often due to virologic failure with the prior regimens, and our current data suggest that individuals with such changes may be more prone to executive dysfunction.

We found no effect of CD4 counts on performance in executive function or verbal fluency tests. These results are different from findings in other settings showing that untreated HIV infection and high viremia were associated with increased risk of neurocognitive impairments^63,64^, and that cART use and viral control lowers the risk neurocognitive dysfunction^64–68^. Studies of HIV+ subjects in other resource-limited countries, including in SSA^67,69,70^, showed that cART use was associated with improved cognitive function, with 6 months to 1 year cART associated with significantly better executive function and verbal fluency in South Africa^69^ and Uganda^70^. Large variations in duration of cART use may have played a role in the discrepancies observed. Whereas all cases in the Ugandan^70^ and South African^69^ studies had respectively been on cART for 6 months and 1 year, HIV+ subjects on treatment in our study had been on cART for a median duration of 3.3 years (IQR: 1.5 to 6 years). A randomized clinical trial of 860 HIV+ subjects from seven resource-limited countries (in SSA, Asia, and South America) who were regularly followed up for 4 years also showed no significant improvement in Category Fluency over 4 years cART, although it showed significant improvements in other cognitive domains such as complex motor function^67^. Our subsequent studies will determine whether ART and viremia affect other neurocognitive domains in HIV+ Cameroonians.

Although HIV does not infect neurons, viral and cell-mediated factors from productively infected CNS cells such as brain macrophages and microglia induce neuronal injury and death, leading to HAND^71–73^. Inflammation plays a major role in HAND pathogenesis^74–76^, and HIV-1 virions, as well as viral proteins such as Tat and gp120, induce the expression and secretion of inflammatory cytokines and chemokines on the human brain endothelium, resulting in endothelial injury and BBB dysfunction, as well as increased infiltration of virions and infected cells into the CNS and neuronal injury^77–80^. We previously showed differential effects of viral genotypes in HIV-1-induced BBB inflammation, with significantly lower levels of inflammatory cytokines and chemokines in primary human brain microvascular endothelial cells exposed to HIV-1 CRF02_AG Tat proteins, compared to cells exposed to subtype B Tat proteins^81,82^. HIV-1 CRF02_AG is the predominant subtype in Cameroon and other West and Central African countries^16–18^. Considering this differential inflammation with HIV-1 CRF02_AG and the fact that increased HIV-induced CNS inflammation increases risk of neurocognitive impairment^74–76^, we explored whether there were differential HIV-1 effects on executive function and/or verbal fluency for subjects infected with CRF02_AG, compared to subjects infected with other HIV subtypes. Analyses showed no difference in verbal fluency based on subtype groups but compared to subjects infected with AG-Plus and non-AG HIV-1, subjects infected with CRF02_AG viruses showed less deficit in CTT2, significantly less deficit in SCWT and on the executive function summary T-score. This suggests that our previous findings of reduced inflammation with HIV-1 CRF02_AG and Tat.AG^81,82^ may also correlate with reduced impairment in executive functioning among subjects infected with CRF02_AG viruses, compared to subjects infected with other HIV subtypes. Our subsequent studies will determine whether there is a correlation between viral genotype, systemic inflammation, and risk of other neurocognitive impairments in these subjects.

## Conclusions

In summary, our current study showed that after adjusting for age, gender and education, there was a significantly higher proportion of HIV+ Cameroonians with impairments in executive functioning and verbal fluency compared to HIV- controls. Also, we found that compared to subjects infected with CRF02_AG viruses, infection with non-AG and AG-Plus subtypes was associated with increased deficits in executive function. Cross-sectionally, cART use, viral loads, and CD4 counts were not associated with NP test scores, but a prospective, longitudinal study would be needed to clarify such effects. Our current study provides normative data in healthy adults Cameroonians (age 18–64) for four NP tests often used to assess executive function (CTT2, HCT, SCWT, and WCST)^25,26^, and three NP tests used to assess verbal fluency (Action Fluency, Category Fluency, and Letter Fluency)^28,29^. These data will be useful reference values for future research and clinical studies assessing impairments in executive function and verbal fluency in Cameroon. These baseline metrics will also facilitate future investigation of diseases and other conditions affecting the brain frontal systems in Cameroon.

## Study Limitations

The subjects recruited here were mostly residents of Yaoundé and its surrounding suburban neighborhoods, which may impact the generalizability of study results. However, Yaoundé, the capital city, is the largest city in Cameroon, with over 3 million inhabitants, and is a cosmopolitan city that includes people from diverse backgrounds and from all Cameroonian ethnic groups^14^. Although our sample size (742 subjects, 395 HIV− and 347 HIV+) was larger than sample sizes in many other studies of HAND in SSA, we observed differences in age, education, and gender distribution between the two groups. Although the use of demographically corrected test scores greatly mitigated these cohort differences, we cannot rule out the possibility that other (unmeasured) background differences may have affected the results (e.g. lower education could be associated with lower socio-economic status, but we did not have data on subjects’ socio-economic status).

## Materials and Methods

### Psychometric instruments

#### Halstead Category test

The Halstead Category test (HCT) used was a computer-based NP test that involves reasoning, abstract thinking, problem-solving, attention, and memory^35^. For HCT, the respondent examines a series of designs projected on a computer screen, to discern underlying principles or themes through trial and error learning and hypothesis testing^35^. For each test item, the respondent indicates their response by pressing the appropriate computer key on the answer panel. Each response is followed by an immediate feedback consisting of a bell ring for a correct answer and a buzzer sound for a wrong answer. HCT is a very sensitive measure of frontal lobe function that includes 7 subtests: subtests I and II evaluate simple recognition of Roman numerals and number counting; subtest III assesses abstract reasoning; subtests IV, V and VI require spatial reasoning, while subtest VII evaluates learning and retention of the concepts associated with other subtests. At the end of the test administration, the software provides performance scores that include the total number of errors, and the number of errors made in each subtest^35^.

#### Wisconsin Card Sorting test-64

Wisconsin Card Sorting Test-64 (WCST-64)^37,39^ is a measure of frontal lobe function that assesses the ability to learn simple concepts and think flexibly. WCST-64 is a computer-based sorting test for a deck of 64 cards, in which the respondent must adapt to changing sorting criteria. The WCST-64 scoring software provides performance scores, including total errors (the summary measure used here).

#### Color Trails-II

The Color Trails-II Test (CTT2) measures attention, mental processing speed, and the ability to mentally control responses to irrelevant aspects of simultaneous stimulus patterns^36^. The Color Trails Test has the sensitivity and specificity of the standard Trail Making Test (Part B) but may be less biased by differences in cultural and linguistic backgrounds, and its validity has been demonstrated in studies involving diverse populations^25^. The CTT2 consists of a sheet with pink and yellow circles numbered 1 to 25, where the respondent alternates between pink and yellow colors to rapidly connect sequentially numbered circles^36^. A stopwatch was used to record each trail completion time.

#### Stroop Color-Word Interference test (SCWT)

The Stroop Color-Word Interference test (SCWT) measures multiple cognitive functions dependent on the frontal lobe integrity, including attention capacity and ability to process and control interference^38^. The Golden version of the SCWT used in this study consisted of 3 pages/3 trails, each with 100 items presented in 5 columns of 20 items. The 1^st^ page/trial consisted of color words “red”, “green”, “blue”, written in black ink (all in upper case letters), with subjects having to read aloud the words “red”, “green”, “blue” from left to right, as quickly as possible for 45 seconds (s). The 2^nd^ page/2^nd^ trial consists of a series of words written in congruent blue, red, or green ink colors (e.g., the word “blue” written in blue ink), and subjects had to name the color of each ink, from left to right, as quickly as possible for 45 seconds. The 3^rd^ page/3^rd^ trial consisted of an interference test, with the words “red”, “green”, “blue” (all in upper case letters) written in a different/incongruent ink from the color word (e.g. “red” written in blue or green ink), and subjects had to name the ink color as quickly as possible for 45 seconds. The SCWT interference scores consisted of the number of items correctly identified during the 3^rd^ trial (executive function trial).

#### Verbal Fluency tests

Verbal fluency tests are often used to evaluate language and cognitive performance associated with frontal lobe function^27–30^. For the present study, tests of verbal fluency (Letter Fluency, Category Fluency, and Action Fluency) were administered as previously described^24,83,84^. Briefly, for Letter Fluency, participants were instructed to generate as many words as possible that begin with the letter “F”, “A”, and “S”, within 60 seconds for each letter category. Participants were instructed to avoid proper names (names of people or places), plurals, or a variation of the same word, and such words, as well as intrusions (words beginning with a different letter) were not counted. The total score consisted of the total number of “F”, “A”, and “S” words correctly generated within the time limits. For Category Fluency, participants were instructed to generate as many animals’ names as possible within 60 seconds. For Action Fluency, participants were instructed to name different things that people do, as many as possible, within 60 seconds. The scores consisted of the total number of correct verbs generated.

#### Training and adaptation of NP tests

The NP tests used in this study are part of the HIV Neurobehavioral Research Center HNRC) international NP test battery that we previously translated into French, and standardized and piloted in Cameroon^84^. NP tests were also back-translated into English, and back-translated tests were similar to the original English version of the tests^84^. This battery has been shown to be sensitive in detecting HAND in several other settings, including in the USA^85,86^, India^87^, China^48,83^, Brazil^88,89^, Cameroon^84^, Nigeria^10,46,52,90^, Zambia^91–93^, and South Africa^51,94^. The NP tests were administered by trained psychometrists. To adapt NP and neuromedical procedures to Cameroon settings and ensure standardization, the Cameroon investigators (JYF, RD, AK, GDK, AKN) were trained and certified by American neuropsychologists and neuromedical personnel (DF, RKH and his team) at the HNRC, University of California San Diego, U.S. At the beginning of the study in Cameroon, quality assurance reviews were conducted by HNRC scientists on test forms of the first 5 visits, and thereafter on randomly selected 5 to 10% of all visits.

#### Study population

A total of 395 healthy HIV seronegative (HIV−) controls and 347 HIV seropositive (HIV+) individuals were recruited between 2008 and 2017. The HIV+ cases were recruited from (1) the HIV voluntary counseling and testing sections of the Day-care Service in the Yaoundé Central Hospital; (2) the Yaoundé Jamot Hospital; (3) the Efoulan District Hospital, Yaoundé; and (4) the Etoug-Ebe Baptist Hospital, Yaoundé. Seronegative controls were recruited from the same health services, as well as among (1) caregivers and visitors to the Neurology outpatient clinic and Day-care service in the Yaoundé Central Hospital; (2) the Health and Social Welfare Centre of the University of Yaoundé−1; and (3) Yaoundé general population. The purpose of the study and research procedures were fully explained to participants and only adults at least 18 years old who did not have exclusion criteria and who gave a written consent were included in the study. The exclusion criteria were: (1) present or past history of CNS disease unrelated to HIV, (2) head trauma, (3) current alcohol intoxication (blood alcohol content of each participant was measured using a Breathalyzer), (4) known psychiatric disease or treatment with antipsychotic drugs, and (5) ongoing systemic illness or fever (temperature of 37.5 °C or higher). All subjects enrolled spoke French as their primary language and interviews and NP testing were thus conducted in French.

#### Data collection

All participants provided demographic information, and underwent a complete medical history, a general physical examination, and a thorough neurological assessment by neurologists at the Yaoundé Central Hospital to detect any focal neurological deficit suggestive of CNS opportunistic infection, before psychometric testing. This thorough clinical assessment of each subject combined with review of his or her prior medical history and subsequent laboratory data, ensured that potential confounding factors such as existing CNS opportunistic infections were ruled out. Executive function (HCT^35^, WCST^37,39^, CTT2^36^, and SCWT^38^) and verbal fluency (Letter Fluency, Category Fluency, and Action Fluency)^24,83,84^ tests were administered to each subject by trained psychometrists in the Neuropsychology Laboratory of the Neurology Department of the Yaoundé Central Hospital, in a private, quiet and well-lit room. Psychometric testing was done prior to blood and urine sample collections and laboratory analyses for HIV serology, CD4 counts, viral loads, and substance use.

#### Norming procedure

Employing the norming methods described in detail by Casaletto and colleagues^40,41^, raw scores of each NP test were converted to uncorrected normalized scaled scores (SS, *M* = 10, *SD* = 3), and these SS were then calculated to T-scores corrected for age, gender, and education, using in-house R scripts. Briefly, scaled scores were obtained by standardizing raw score quantiles and scaling them with a mean of 10 and standard deviation of 3. Scaled scores were then approximated as a function of age, gender, and education by fitting a multivariable fractional polynomial (MFP) model^95^, using R package mfp (https://cran.r-project.org). The MFP model searches for an appropriate transformation of numeric covariates and considers non-linear relationships of covariates with the outcome. Stability of the MFP curves were checked through the sensitivity analyses for the MFP models using bootstrap procedure (K = 1000)^96^. The residuals obtained from the MFP model were standardized and converted into T-scores. The demographically corrected T-scores have a mean score of 50 and standard deviation of 10. T-scores for the HIV+ cohort were calculated from the formulas developed on the normative group. For each test, T-scores were converted to deficit scores to assess the degree of impairment (from 0 = no impairment [T = 40+] to 5 = severe impairment [T < 20]). Domain T-scores and deficit scores represent the averaged values from the individual tests. “Impairment” criteria were domain average deficit scores of >0.50, which ensure that, on average, the participant was at least mildly impaired on more than half of the test measures.

#### HIV serology, CD4 cell counts, and viral loads

Sample collection and all analyses were performed in the Hematology laboratory of the Yaoundé University Teaching Hospital or the International Reference Centre Chantal Biya, Cameroon. Venous blood samples were collected and stored at room temperature in the outpatient clinic and analyses performed in the Hematology laboratory within 6 hours of blood collection. The HIV status of each participant was determined using the rapid immunochromatographic HIV-1/2 test (Abbott Diagnostics, Chicago, IL, USA) and the Murex HIV antigen/antibody Combination ELISA (Abbott Diagnostics), according to the manufacturer’s instructions. A participant was considered HIV+ if he/she tested positive for the two tests, HIV− if negative for both tests, and discordant if positive for only one test. No discordant result was observed in this study.

Subjects’ CD4 T-lymphocyte counts were quantified by flow cytometry, using a Fluorescence Activated Cell Sorting (FACS) Count Instrumentation System and the BD FACSCount CD4 reagent kit (BD Biosciences, San Jose, CA, USA), according to the manufacturer’s instructions. The FACS instrument was calibrated and quality control tested before each assay. For viral load determination, HIV RNA copy number in each plasma sample was quantified by reverse transcription polymerase chain reaction (RT-PCR), using Amplicor HIV-1 Monitor Test (Roche Diagnostic Systems, Pleasanton, CA), according to the manufacturer’s protocol. The assay detection limit was 50 viral copies/mL.

#### HIV amplification, sequencing and genotyping

Viral RNA was extracted from plasma samples using the QIAmp viral RNA Mini kit (Qiagen Inc., Valencia, CA, USA), according to the manufacturer’s protocol. Extracted RNA (150 to 1200 ng) were reverse transcribed and amplified using a nested PCR with SuperScript One-Step RT-PCR reverse transcriptase and Platinum Taq DNA polymerase (Life Technologies, Carlsbad, USA), according to the manufacturer’s instructions. HIV PR, RT, gag, env (C2V3), tat, and nef genes were amplified as we previously described^17,18^. Primers sequences and reactions conditions are detailed in our previous publications^17,18^. Amplicons were purified, sequenced at the University of Nebraska Medical Center High-Throughput DNA Sequencing and Genotyping Core Facility, and nucleotide sequences analyzed as we previously described^17,18^.

#### Statistical analyses

Demographic data were compared between controls and HIV+ subjects using Student’s *t*-tests for continuous variables and Fisher’s exact test for binary variables. Univariable analysis was performed to examine the associations of T-scores for executive function tests (Color Trails-II Time, HCT Total Errors, SCWT and WCST Total Errors) and for verbal fluency tests (Category Fluency Trial 1 Words, Action Fluency, and Letter Fluency) with demographic factors (age, gender, and education) in controls and HIV+ subjects separately. The associations of raw scores for executive function and verbal fluency tests with demographic factors were also assessed. T-scores were compared between the two groups (HIV+ vs. HIV−). The proportions of neurocognitive impairment in executive function and verbal fluency tests (impaired if individual test deficit score ≥1) and domains (impaired if domain deficit score >0.5) were then compared between controls and HIV+ subjects using logistic regression. Additionally, in HIV+ participants, T-scores were compared between treatment naïve and patients on cART, patients with higher (≥350) and low (<350) CD4 cell counts, and patients with undetectable (<50 copies/mL) and detectable (≥50 copies/mL) HIV RNA viral loads, as well as HIV subtypes. For HIV+ patients on cART, three separate comparisons of cognitive scores were made between persons on regimens that do and do not contain NVP, between persons on regimens that do and do not contain ZDV, and between persons with a history of one and multiple regimens. R software (version 3.4.1) was used to perform statistical analyses. Results were considered statistically significant at a p-value of less than 0.05. Cohen’s d effect sizes (and 95% confidence intervals) were reported for the differences between groups.

### Ethical approval and informed consent

This study was conducted in accordance with the Declaration of Helsinki. The study protocol was approved by the Cameroon National Ethics Committee, as well as the Institutional Review Board of the University of Nebraska Medical Center. All subjects gave written informed consent for inclusion before participating in the study.

## Data Availability

Nucleotide sequences for clinical isolates reported in this study are available in the NCBI database; Genbank accession numbers included in our previous publications^17,18^.
